# Advances in translational biomedicine from systems approaches

**DOI:** 10.3389/fgene.2014.00273

**Published:** 2014-08-14

**Authors:** Enrico Capobianco, Pietro Lió

**Affiliations:** ^1^Center for Computational Science, University of MiamiMiami, FL, USA; ^2^Laboratory of Integrative Systems Medicine, Institute of Clinical Physiology, CNRPisa, Italy; ^3^Computer Laboratory, Cambridge UniversityCambridge, UK

**Keywords:** systems biomedicine, translational science, big data, inference, paradigm shift

Systems Biomedicine (see for instance Antony et al., 2012) is a field in perpetual development. By definition a translational discipline almost holistically centered on the patient, it emphasizes in light of its multifaceted characterization the need of assessing its constitutive components as a system, whose dynamics occur across multiple and hierarchical scales (organs, tissues, cells, molecules).

A principal role in systems approaches is played by quantitative inference methods resolving problems of high complexity and uncertainty levels. Not surprisingly, it is expected that complex systems may generate information from heterogeneity of sources and diversity of components. Researchers can use this information to look for data patterns with a signal-to-noise ratio which help explaining variation and interdependent phenomena (gene expression and methylation, pervasive transcription, alternative splicing etc.).

Next-Gen technologies and Electronic Medical/Health Records are providing researchers with data resources correctly classified as “Big Data” (Pathak et al., 2013). The management of such resources implies that a cross-disciplinary approach must be put in place, involving team work targeting multiplexed research topics (clinical, experimental, omics, high-content imaging, etc.) whose separate analysis would not be as informative as their synergistic fusion. In parallel, the growing impact of integration of medical records, epidemiological studies and quantitative measures referred to patients is increasingly expanding the frontier of personalized or individualized medicine by leveraging on a multi-evidenced mosaic of information designed to improve patient-specific profiling.

While in principle it clearly appears from the most recent literature what systems biomedicine is aiming to achieve, and the attention is now on what instruments are needed, a main question to pose is: *How fast and effectively are we moving into this translation?* Given the current speed at which the translation is taking place, there are cultural, technical and methodological bottlenecks that need be solved. The proposed Special Topic on “Comprehensive Systems Biomedicine” overviews the path of progression of the field along three main axes:

1. Data: once the accessibility is guaranteed and the dimensionality is managed, these will require novel generation analytics to discriminate between signal and noise and thus reveal with accuracy the inherent verifiability, relevance, completeness, prediction power making of the data optimal candidate for integrative inference approaches. The non-coding RNA role is being increasingly revealed by high-throughput studies (The ENCODE Project Consortium, 2004; Harrow et al., 2012) in both healthy and diseased conditions, but refers also to the possibility of re-using data from previous technologies, i.e., microarray, as shown by the contributed work on *neuropathic pain*. Then, this role is destined to have a strong impact in *pluripotency and neural differentiation of hESCs and ihPSCs* (following Li et al., 2011). Also, data integration is currently a major topic, in particular with reference to *profiling and pathway annotation of large-scale cancer cell lines*.
2. Methods: when modularly designed and semi-parametric, methods guarantee wide-spectrum applicability. Hybrid pipelines can take advantage of different quantitative approaches (statistics, machine learning, optimization, control, graph theory) combining multiple platform outcomes, with analyzers and optimizers outflowing into metadata and visual frameworks. *Molecular interaction network approaches in pharmacology* are reviewed in a contributed study, while in another study *magnetic resonance techniques* are discussed with regard to morphological and physiological characterizations of cancer tissue *in vivo*. Finally, a study is presented for *pathway, network, and multiplex methods in the context of brain data*.
3. Systems: an organized functionally interactive aggregate of entities operating under coordinated and harmonic rules in normal conditions, should be comparatively evaluated against altered (disordered, dysregulated, etc.) conditions to assess phenotypic variations determining the systems characteristics preventively or prospectively, at disease onset and pre/post intervention. In one example, the integration of cytokines, lipoproteins, tissue proteins, and histology indexes cast within a statistical model to study plaque growth opens for new possible interpretations of the *atherogenesis inflammatory disorder.* In another study, the *modeling of metabolism* is considered and an algorithm proposed to detect functional groups from existing databases and to identify metabolites, and from the perspective of *pandemic studies*. Then, a study introduces an *information system for precision oncology* designed for the integration of data and real-time processing of samples with the computational analysis of genomic alterations and mutations observed in the molecular profiles.

The three axes—Data, Methods, Systems—can be naturally integrated through key properties (such as compatibility, transferability, generalizability), characteristic features and state-of-the-art tendencies.

The communication across the axes is established on the basis of the specific application domains. The final impacts (clinical, societal, etc.) depend on both axis prioritization and solutions that are selected to optimize the key properties.

While much work is on the way for empowering systems approaches to enable a change in biomedical research, we hope that the newly presented studies in this Special Topic can offer opportunities to appreciate the current endeavors and prospective potential in this field.

## Conflict of interest statement

The authors declare that the research was conducted in the absence of any commercial or financial relationships that could be construed as a potential conflict of interest.
